# Supplementary material for: Early pandemic associations of latitude, sunshine duration, and vitamin D status with COVID-19 incidence and fatalities: A global analysis of 187 Countries
Source: PLOS Glob Public Health. 2025 Jul 28;5(7):e0004074. doi: 10.1371/journal.pgph.0004074 (PMC12303322; doi:10.1371/journal.pgph.0004074)
Supplement: S1 Table — (DOCX) [file pgph.0004074.s001.docx]

**S1 Table. Effect of latitude, amount of sunshine and vitamin D status on COVID-19 prevalence, mortality rate and case fatality rate by March 31^st^ and September 30^th^ 2020**

|  |  |  | **Latitude** | | |  | **Sunshine** | | |  | **Vitamin D** | | | |  |
| --- | --- | --- | --- | --- | --- | --- | --- | --- | --- | --- | --- | --- | --- | --- | --- |
|  |  |  | **n** | **Beta (95% CI)** | ***p*** |  | **n** | **Beta (95% CI)** | ***p*** |  | **n** | **Beta (95% CI)** | ***p*** |  |  |
| **March 31^st^** | Prevalence^a^ | Univariate | 182 | 0.058 (0.036, 0.080) | <0.0001 |  | 128 | -4.041 (-5.075, -3.007) | <0.0001 |  | 76 | -0.039 (-0.081, 0.002) | 0.061 |  |  |
|  |  | Multivariate^*^ | 165 | 0.020 (0.004, 0.035) | 0.012 |  | 121 | -0.656 (-1.545, 0.234) | 0.15 |  | 74 | -0.036 (-0.057, -0.014) | 0.002 |  |  |
|  | Mortality rate^b^ | Univariate | 182 | 0.079 (0.048, 0.109) | <0.0001 |  | 128 | -5.010 (-6.850, -3.171) | <0.0001 |  | 76 | -0.080 (-0.134, -0.026) | 0.004 |  |  |
|  |  | Multivariate^*^ | 165 | 0.033 (0.003, 0.063) | 0.034 |  | 121 | -0.232 (-2.395, 1.931) | 0.83 |  | 74 | -0.073 (-0.119, -0.027) | 0.002 |  |  |
|  | Case fatality rate (%)^c^ | Univariate | 182 | 0.028 (0.003, 0.053) | 0.027 |  | 128 | 4.389 (-0.758, 9.536) | 0.094 |  | 76 | -0.041 (-0.080, -0.002) | 0.042 |  |  |
|  |  | Multivariate^*^ | 165 | 0.017 (-0.011, 0.046) | 0.24 |  | 121 | -1.269 (-6.730, 4.192) | 0.65 |  | 74 | -0.037 (-0.080, 0.005) | 0.086 |  |  |
| **September 30^th^** | Prevalence^a^ | Univariate | 182 | 0.014 (-0.004, 0.031) | 0.12 |  | 128 | -0.859 (-2.308, 0.589) | 0.24 |  | 76 | -0.021 (-0.042, 0.000) | 0.045 |  |  |
|  |  | Multivariate^*^ | 165 | 0.005 (-0.007, 0.017) | 0.42 |  | 121 | 1.005 (-0.388, 2.398) | 0.16 |  | 74 | -0.024 (-0.044, -0.004) | 0.019 |  |  |
|  | Mortality rate^b^ | Univariate | 182 | 0.025 (0.001, 0.044) | 0.042 |  | 128 | -2.203 (-4.566, 0.161) | 0.067 |  | 76 | -0.052 (-0.085, -0.020) | 0.002 |  |  |
|  |  | Multivariate^*^ | 165 | 0.013 (-0.012, 0.039) | 0.30 |  | 121 | 0.415 (-1.897, 2.727) | 0.72 |  | 74 | -0.052 (-0.086, -0.018) | 0.003 |  |  |
|  | Case fatality rate (%)^c^ | Univariate | 184 | 0.010 (-0.007, 0.026) | 0.26 |  | 130 | -0.947 (-2.614, 0.720) | 0.26 |  | 76 | -0.031 (-0.057, -0.006) | 0.017 |  |  |
|  |  | Multivariate^*^ | 167 | 0.008 (-0.011, 0.028) | 0.38 |  | 122 | -0.415 (-2.331, 1.501) | 0.67 |  | 74 | -0.028 (-0.056, 0.000) | 0.051 |  |  |
| n ;number of countries included in the analyses ^a^COVID-19 prevalence as total number of reported cases per 1 million of population, ^b^COVID-19 mortality rate as number of deaths per 1 million of the population and ^c^COVID-19 case fatality rate as number of deaths/number of reported cases x 100. *Multivariate model was adjusted for gross domestic product (GDP), elderly dependency ratio (EDR) and population density. | | | | | | | | | | | | | | | |
