# Supplementary figures and images for: Early pandemic associations of latitude, sunshine duration, and vitamin D status with COVID-19 incidence and fatalities: A global analysis of 187 Countries
Source: PLOS Glob Public Health. 2025 Jul 28;5(7):e0004074. doi: 10.1371/journal.pgph.0004074 (PMC12303322; doi:10.1371/journal.pgph.0004074)

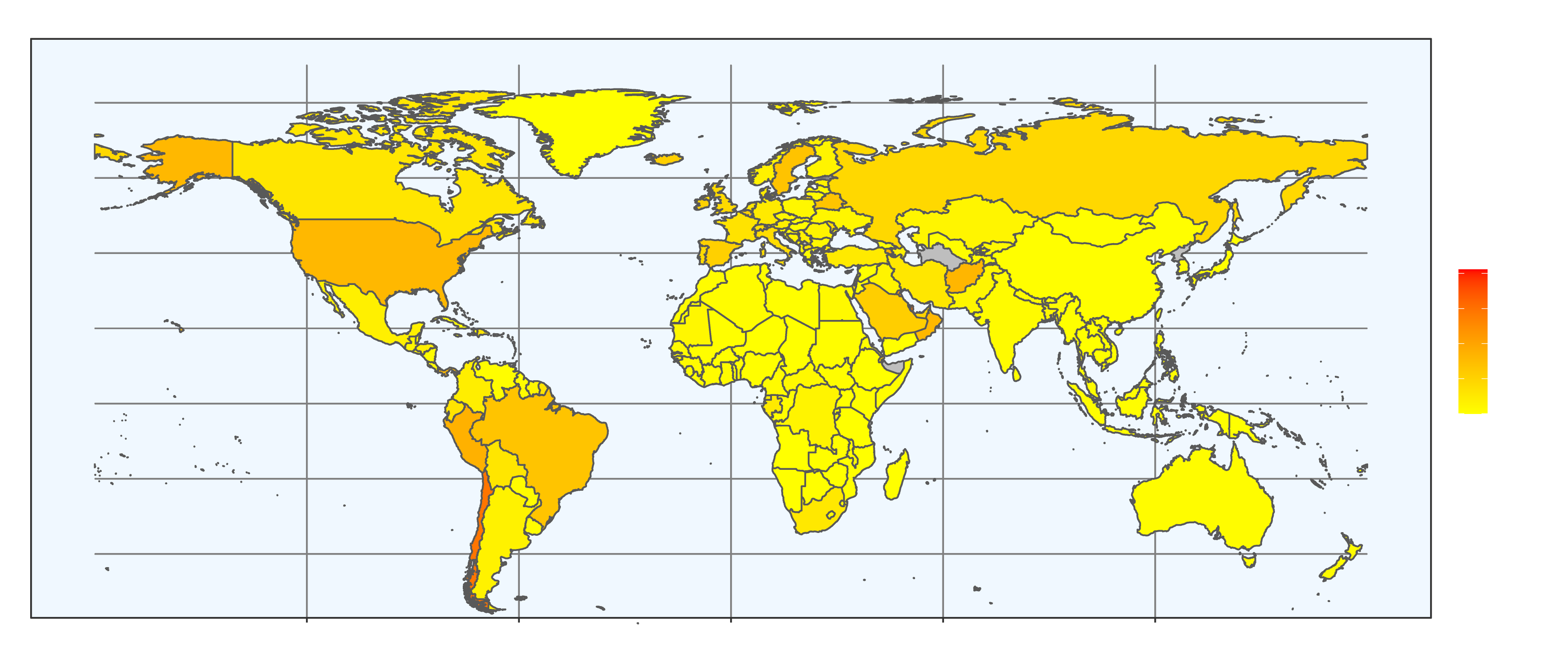

Supplement: S1 Fig — (TIF) [file pgph.0004074.s002.tif]
